# Global distribution of functionally important *CYP2C9* alleles and their inferred metabolic consequences

**DOI:** 10.1186/s40246-023-00461-z

**Published:** 2023-02-28

**Authors:** Yitian Zhou, Lenka Nevosadová, Erik Eliasson, Volker M. Lauschke

**Affiliations:** 1 Division of Clinical Pharmacology, Department of Laboratory Medicine, Karolinska Institutet, 141 52 Huddinge, Sweden; 2 Department of Physiology and Pharmacology, Karolinska Institutet, 171 77 Stockholm, Sweden; 3 Medical Diagnostics, Clinical Pharmacology, Karolinska University Hospital, 141 86 Stockholm, Sweden; 4 Dr Margarete Fischer-Bosch Institute of Clinical Pharmacology, Stuttgart, Germany; 5 University of Tübingen, Tübingen, Germany

**Keywords:** Pharmacogenomics, Precision medicine, Allele frequency, Metabolizer phenotype, Precision public health

## Abstract

**Background:**

Genetic variability in the cytochrome P450 CYP2C9 constitutes an important predictor for efficacy and safety of various commonly prescribed drugs, including coumarin anticoagulants, phenytoin and multiple non-steroidal anti-inflammatory drugs (NSAIDs). A global map of *CYP2C9* variability and its inferred functional consequences has been lacking.

**Results:**

Frequencies of eight functionally relevant *CYP2C9* alleles (*2, *3, *5, *6, *8, *11, *13 and *14) were analyzed. In total, 108 original articles were identified that included genotype data from a total of 81,662 unrelated individuals across 70 countries and 40 unique ethnic groups. The results revealed that CYP2C9*2 was most abundant in Europe and the Middle East, whereas CYP2C9*3 was the main reason for reduced CYP2C9 activity across South Asia. Our data show extensive variation within superpopulations with up to tenfold differences between geographically adjacent populations in Malaysia, Thailand and Vietnam. Translation of genetic *CYP2C9* variability into functional consequences indicates that up to 40% of patients in Southern Europe and the Middle East might benefit from warfarin and phenytoin dose reductions, while 3% of patients in Southern Europe and Israel are recommended to reduce starting doses of NSAIDs.

**Conclusions:**

This study provides a comprehensive map of the genetic and functional variability of CYP2C9 with high ethnogeographic resolution. The presented data can serve as a useful resource for *CYP2C9* allele and phenotype frequencies and might guide the optimization of genotyping strategies, particularly for indigenous and founder populations with distinct genetic profiles.

**Supplementary Information:**

The online version contains supplementary material available at 10.1186/s40246-023-00461-z.

## Introduction

Inter-individual variability in drug response that leads to either adverse drug reactions (ADRs) or low drug efficacy is commonly observed in clinical practice and poses significant burden to patient health and health care system. Insufficient drug efficacy and drug-related toxicity occur in up to 50% of the clinical treatments and ADRs accounts for 6–9% of all hospital admission, of which up to 40% are life threatening [1–3]. Importantly, it is estimated that 20–30% of the variability in drug response can be explained by genetic polymorphisms that are primarily localized in genes involved in drug absorption, distribution, metabolism and excretion (ADME), as well as in drug target genes and immune-related genes [4].

The human cytochrome P450 (CYP) superfamily, comprises 57 functional genes and constitutes the largest family of enzymes involved in phase I drug metabolism [5]. Among them, CYP2C9 is the most abundantly expressed CYP2C isoform in the liver and accounts for around 20% of the hepatic CYP proteins quantified by mass spectrometry [6]. It metabolizes a variety of commonly prescribed drugs, including coumarin anticoagulants, NSAIDs and sulfonylureas, as well as endogenous substrates, such as arachidonic acid [7, 8]. Genetic polymorphisms in *CYP2C9* have long been recognized as a determinant of inter-individual CYP2C9 variability. The most well-studied *CYP2C9* alleles are CYP2C9*2 (NC_000010.11:g.94942290C > T, p.R144C, rs1799853) and *3 (NC_000010.11:g.94981296A > C, p.I359L, rs1057910). In vitro, CYP2C9*2 reduces enzyme activity by 50–70% whereas CYP2C9*3 almost completely abrogates enzyme function (reduction of 75–99%) [9, 10]. Both alleles have also been associated with decreased metabolism of many CYP2C9 substrates in vivo, including S-warfarin and phenytoin [11]. Besides *2 and *3, multiple other variant alleles can affect CYP2C9 activity, including the decreased function alleles CYP2C9*5 (NC_000010.11:g.94981301C > G, p.D360E, rs28371686), *8 (NC_000010.11:g.94942309G > A, p.R150H, rs7900194), *11 (NC_000010.11:g.94981224C > T, p.R335W, rs28371685), *14 (NC_000010.11:g.94942234G > A, p.R125H, rs72558189) and the loss-of-function (LOF) alleles *6 (NC_000010.11:g.94949283del, p.Lys273fs, rs9332131) and *13 (NC_000010.11:g.94941958 T > C, p.L90P, rs72558187) [12]. Due to the significant impact of *CYP2C9* variations, the US Food and Drug Administration (FDA) and the European Medicines Agency (EMA) include *CYP2C9* genotyping in the drug labels or summary of product characteristics of 19 drugs. Specifically, testing is required for the sphingosine-1-phosphate receptor modulator siponimod in multiple sclerosis and *CYP2C9* genotype is also considered as actionable information for dosage of warfarin, phenytoin and several non-steroidal anti-inflammatory drugs (NSAIDs) [13].

Considerable variation in *CYP2C9* allele frequencies across different populations and ethnicities has been observed. Previous studies reported that CYP2C9*2 was most abundant in European populations (minor allele frequency, MAF between 11.1 and 14.4%), whereas the highest frequency of CYP2C9*3 was found in Asians (MAF up to 13%) [14–17]. However, frequencies were commonly extrapolated from subpopulations within the same geographic group and the generalizability of these results remains questionable [14]. In addition, evaluation of *CYP2C9* allele frequencies with higher resolution is required to account for the complex patterns of ethnogeographic variability that are not reflected when only aggregated populations are considered. To better understand the global distribution of CYP2C9 variability, we here systematically analyzed *CYP2C9* allele frequency data from the literature, covering in total 81,662 unrelated individuals across 70 countries and 40 ethnogeographic groups. In addition, we translate these allele frequencies into functional metabolic consequences, thus providing the first comprehensive overview of genetic and inferred functional variability at a global scale.

## Results

### Geographic distribution of functionally important CYP2C9 alleles

**Fig. 1:**
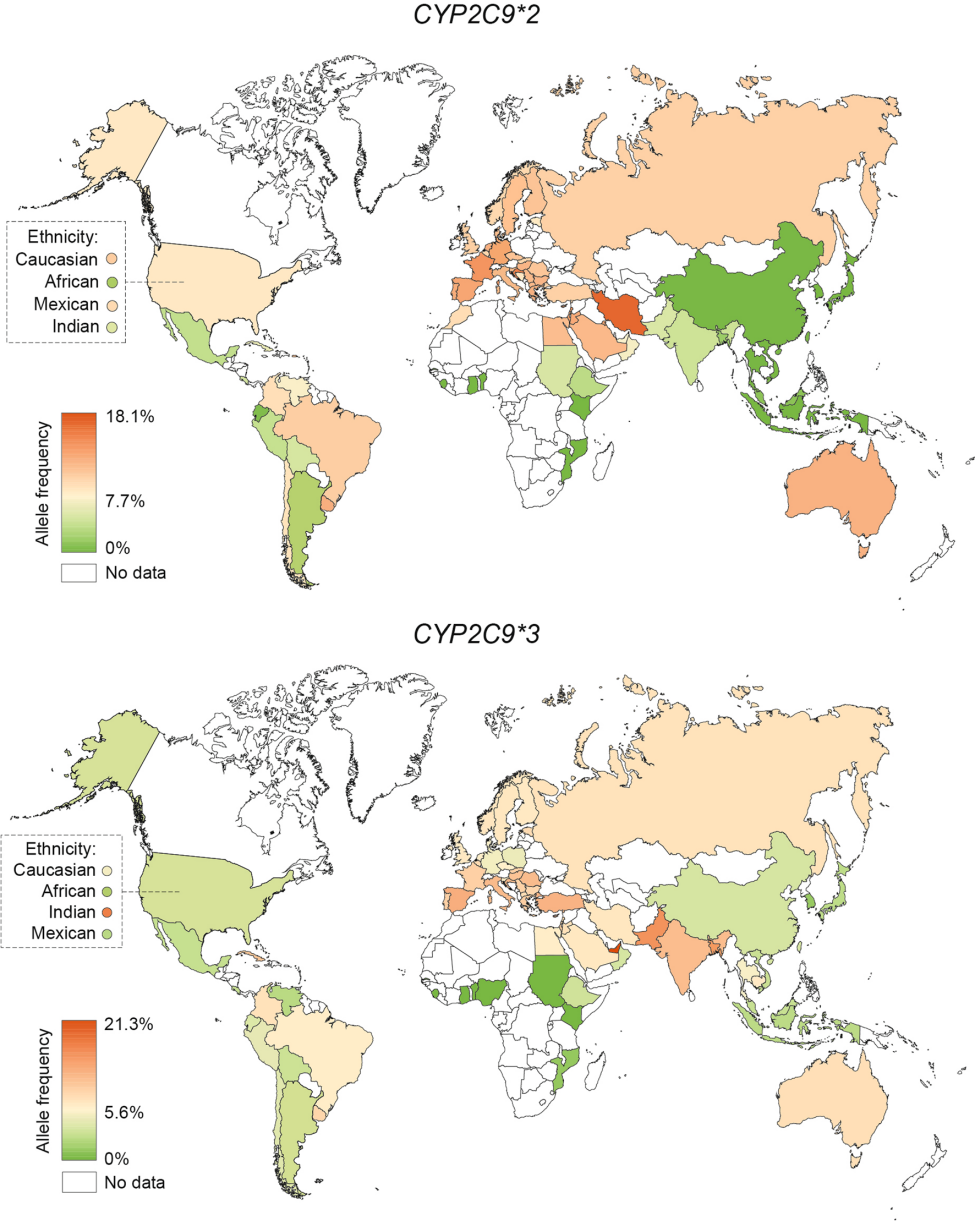
Global distribution of CYP2C9*2 and *3 alleles. Frequencies of 65 countries were color-coded with the highest frequency in red, the average frequency across all countries in yellow, and the lowest frequency in green. Countries with no frequency information available are colored white

Analysis of the frequencies of functionally relevant *CYP* alleles across 70 countries showed that CYP2C9*2 was most abundant in Europe and across the Middle East (Fig. 1; Table 1). The prevalence was overall highest in Iran (minor allele frequency; MAF = 18.1%), followed by Croatia (MAF = 16.5%), Lebanon (MAF = 15.4%) and France (MAF = 15%). In contrast, CYP2C9*2 was absent in East Asian populations and low in South Asia with frequencies pivoting around 5%. In Africa, CYP2C9*2 was generally absent in Sub-Saharan Africa, but relatively high in North African populations (up to 12%). Notably, data about the genetic variability of *CYP2C9* is still lacking for many African countries, suggesting that further exploration in these ethnogeographic groups remains to be important. In the Americas, high CYP2C9*2 frequencies were observed in Brazil (10.7%), but not in Ecuador (0.5%), Mexico (3.7%) and Peru (3.8%).

**Table 1:** National allele frequencies of CYP2C9*2 and *3

Country	N (individuals)	*2 (in %)	*3 (in %)
**Europe**			
*South Europe*			
Bosnia and Herzegovina	81	9.0	-
Croatia	200	16.5	9.5
Greece	283	12.9	8.1
Italy	792	12.4	9.4
Portugal	135	13.2	8.0
Serbia	716	12.3	7.9
Spain	596	13.8	10.1
Turkey	584	10.5	9.8
*North Europe*			
Denmark	276	12.1	5.3
Estonia	2411	8.4	7.2
Finland	12,665	11.4	6.3
Norway	309	9.9	6.5
Sweden	13,495	11.7	6.5
*Central and East Europe*			
Bulgaria	1503	12.5	7.5
Czech	341	11.6	5.9
Germany	118	14.0	5.0
Hungary	535	12.5	8.8
North Macedonia	308	12.4	7.1
Poland	85	-	4.7
Romania	332	11.3	9.3
Russia	290	10.5	6.7
Slovak	112	10.0	8.0
Slovenia	129	12.2	6.3
*West Europe*			
Belgium	121	10.0	7.4
France	151	15.0	8.0
Netherlands	1252	13.0	6.0
UK	397	10.2	5.7
**Africa**			
Benin	111	0.0	0.0
Egypt	247	12.0	6.0
Ethiopia	239	3.3	3.2
Gambia	179	0.4	0.0
Ghana	204	0.0	0.0
Morocco	290	9.5	-
Mozambique	103	0.0	1.0
Nigeria	293	0.0	0.0
Sudan	383	5.0	0.0
**Middle East**			
Iran	693	18.1	6.5
Israel	438	14.3	8.2
Jordan	263	13.5	6.8
Kuwait	108	14.0	5.0
Lebanon	176	15.4	7.8
Oman	641	7.2	3.5
Saudi Arabia	323	12.4	6.3
United Arab Emirates	160	7.2	21.3
**Asia**			
*Southeast Asia*			
Cambodia	122	-	6.6
Indonesia	412	0.0	2.4
Malay	565	1.0	2.9
Thai	1205	0.1	5.3
Vietnam	381	0.0	3.0
*East Asia*			
Bhutan	443	0.3	0.0
China	894	0.1	3.5
Japan	2105	0.0	2.4
Korea	574	0.0	1.1
*South Asia*			
Bangladesh	144	1.7	11.6
India	2435	4.5	9.7
Pakistan	1762	5.0	11.9
**South America**			
Argentina	101	2.6	3.0
Bolivia	778	4.8	3.0
Brazil	721	10.7	6.0
Chile	279	9.0	4.0
Columbia	375	9.3	6.8
Ecuador	297	0.5	3.7
Peru	340	3.8	4.4
Uruguay	103	13.0	7.6
Venezuela	680	7.4	2.3
**North America**			
Costa Rica	443	4.7	2.8
Cuba	128	6.0	9.0
Mexico	1378	3.7	2.8
USA	1411	9.0	4.4
**Oceania**			
Australia	5408	12.8	6.9

Global frequency distributions of CYP2C9*3 align with patterns of CYP2C9*2. European and Middle Eastern populations feature high CYP2C9*3 frequencies particularly in Spain (10.1%) and Turkey (9.8%), whereas the allele was absent or rare in Sub-Saharan Africa and East Asia. A recent study furthermore revealed very high frequencies of CYP2C9*3 in the United Arab Emirates (21.3%), which is in stark contrast to other Middle Eastern populations where CYP2C9*3 frequencies pivot around 6%. In contrast to CYP2C9*2 however, CYP2C9*3 was very common in South Asia with frequencies as high as 11.9% in Pakistan and 11.6% in Bangladesh. In South America, CYP2C9*3 frequencies are relatively higher in Uruguay (7.6%), Columbia (6.8%) and Brazil (6%) but lower than 5% in all other reported countries. Notably, frequency data for Australia was mostly derived from a pan-ethnic Australian population (n = 2,509), which results in an overall close alignment with data from European populations. In contrast, the indigenous Australian Tiwi population exhibited very high frequency of CYP2C9*3 (36%) whereas CYP2C9*2 was absent.

Besides the variants defining CYP2C9*2 and *3, more than 700 additional *CYP2C9* variant alleles have been described, of which approximately 40% have been estimated to impact gene function [18]. While the vast majority of these variants are very rare and their ethnogeographic distribution has not been investigated, the frequencies of six additional functionally relevant *CYP2C9* variant alleles (*5, *6, *8, *11, *13 and *14) have been investigated in multiple populations (Additional file 1: Table S1). Overall, the six alleles were observed in 16 populations, of which *8 and *11 were prevalent in African and South American populations with highest frequency found in Mozambican (14.6%) and Guarani populations (4.4%), respectively. *13 was identified in East Asia and African Americans with frequencies between 0.4 and 1.5%, whereas *5 and *6 were most abundant in the United Arab Emirates (7.8%) and Sudan (2%), respectively.

### Frequencies of CYP2C9*2 and CYP2C9*3 across ethnic groups

In addition to geographic patterns, we analyzed CYP2C9*2 and CYP2C9*3 frequencies across 40 ethnic groups (Table 2). CYP2C9*2 was high in Sephardi Jews (MAF = 19.4%), a Jewish diaspora population originating from the Iberian Peninsula, as well as in Ashkenazim (MAF = 13.5%) who are of Middle Eastern origin with evidence of European admixture [19]. CYP2C9*2 prevalence was very high in Kosovars (MAF = 17.5%), whereas frequencies in neighboring Serbian (MAF = 12.3%) and North Macedonian (12.4%) populations were considerably lower. While CYP2C9*2 is mostly absent or rare in South and East Asia, specific subpopulations, such as Uyghurs (MAF = 7.8%) from Northwest China and the Kannadiga ethnic group (MAF = 6%) from southwest India, feature considerably high frequencies.

**Table 2:** Allele frequencies of CYP2C9*2 and *3 across 35 ethnogeographic groups

Ethnic group	N (individuals)	*2 (in %)	*3 (in %)
**Europe**			
Faroese	312	8.8	5.3
Hungarian Roma	465	11.8	15.5
Kosovar	234	17.5	10.9
Slav	479	10.0	12.0
**Africa**			
Luhya in Webuye, Kenya	120	0.0	0.0
Mende in Sierra Leone	128	0.0	0.0
**Middle East**			
Ashkenazi Jewish	5775	13.5	8.4
Baloch	110	11.8	7.3
Greek Cypriot	148	16.2	11.2
Khorasan	120	9.1	10.0
Iranian Persian	110	11.0	6.0
Sephardi Jewish	80	19.4	14.4
Sistani	140	16.1	7.8
Turkmen	110	8.0	4.0
**Asia**			
*Southeast Asia*			
Jahai people	155	0.0	36.2
*East Asia*			
Mongolian in China	280	0.0	3.0
Tibetan	96	0.0	5.7
Uygur	96	7.8	0.0
*South Asia*			
Andhra Pradesh	116	4.0	9.0
Karnataka	110	6.0	8.0
Kerala	120	2.0	8.0
Tamilian	270	2.8	6.9
**South America**			
Guarani	90	1.1	0.0
**North America**			
African American	982	2.1	1.7
American from India	109	4.9	13.1
American from Mexico	71	10.2	2.3
European American	249	11.2	5.1
Huichol	73	0.0	0.0
Inuit from Canada	151	0.0	0.0
Mestizo from Chile	253	6.0	4.0
Mestizo from Ecuador	297	0.5	3.7
Mestizo from Mexico	947	5.1	3.9
Mestizo from Peru	218	4.6	6.2
Nahua	212	0.7	0.4
Native Indian from Canada	153	3.0	6.0
Puerto Rican	314	12.1	4.3
Teenek	98	0.5	0.5
**Oceania**			
Maori	60	1.7	0.8
Polynesian	1072	3.1	1.6
Tiwi	187	0.0	36

For CYP2C9*3, highest frequencies (MAF = 36.2%) were found in the Jahai people, an indigenous population living in Malaysia. This prevalence is higher than in any other population analyzed and is in stark contrast to national CYP2C9*3 frequencies in Malaysia (2.9%) and other Southeast Asian countries, such as Indonesia (2.4%), Vietnam (3%) and Thailand (5.3%), demonstrating the importance to consider ethnic backgrounds in addition to geographic factors for pharmacogenomic mapping studies.

### CYP2C9 phenotype distribution across different countries and regions

**Fig. 2:**
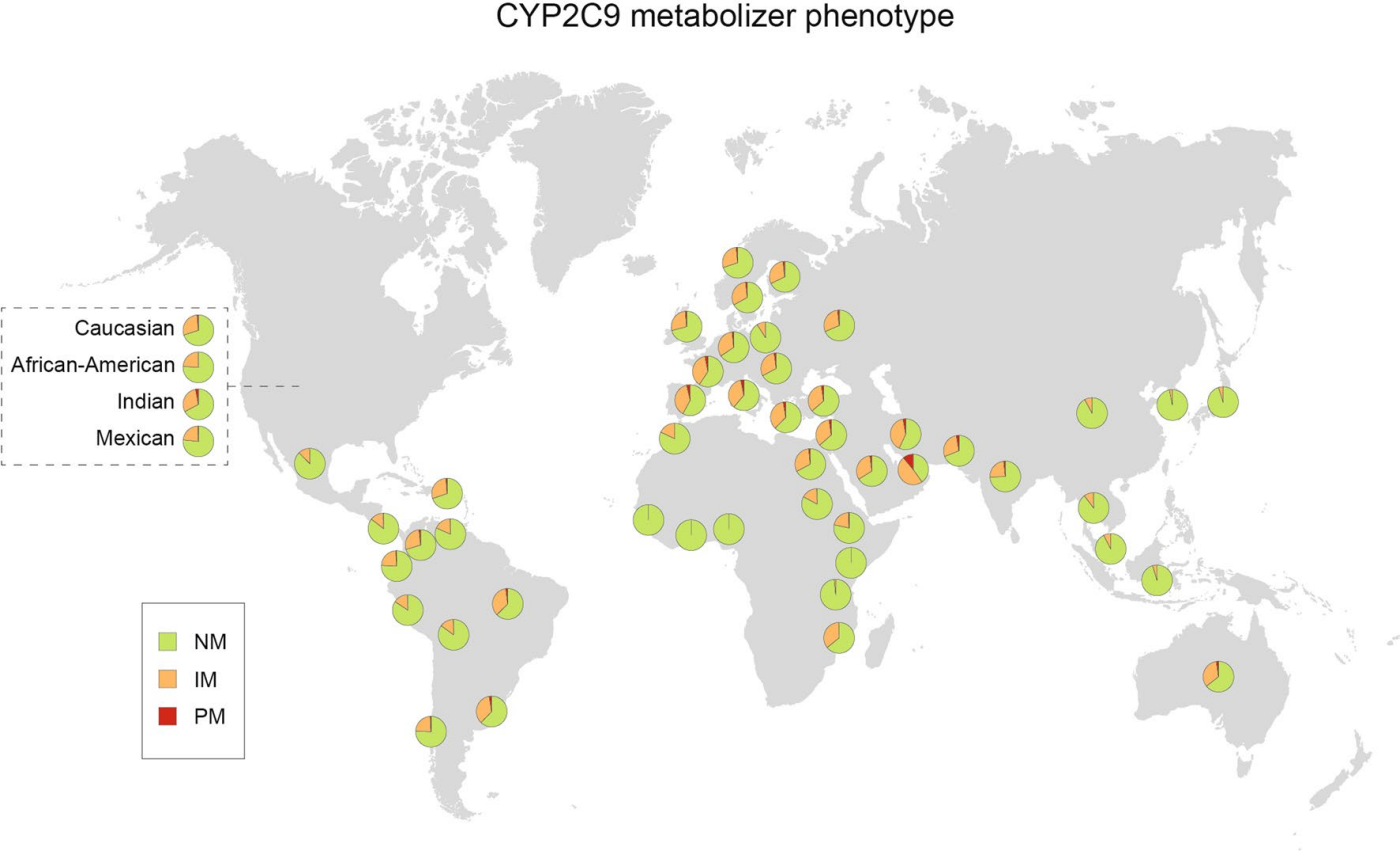
CYP2C9 metabolizer phenotype across different countries and regions. Pie charts illustrate the percentage of normal metabolizer (NM, in green), intermediate metabolizer (IM, in orange) and poor metabolizer (NM, in red) for representative countries

Next, we extrapolated the functional consequences of the observed genetic differences by inferring CYP2C9 metabolic phenotypes based on the frequencies of functionally important *CYP2C9* alleles. To this end, we considered CYP2C9*2 and *3, as well as the less common variant alleles *5, *6, *8, *11, *13 and *14 that decrease or abolish enzyme function. Globally, the prevalence of CYP2C9 poor metabolizers (PMs) is relatively low, ranging from 3–4% in Southern Europe and the Eastern Mediterranean coast to < 1% in Asian and African populations, with the exception of Emiratis (11.1%) due to high frequencies of CYP2C9*3 and *5 (Table 3; Fig. 2). Similar patterns were found for CYP2C9 intermediate metabolizers (IMs), which were overall most common in the United Arab Emirates (48.7%), Croatia (41.2%) and Iran (40.3%) where almost every second individual was estimated to exhibit reduced CYP2C9 metabolism. In Africa where the prevalence of CYP2C9 IM is generally low, we found that around 35.5% were IMs in Mozambique due to the exceptionally high frequency of the decreased function allele CYP2C9*8 in this population (MAF = 14.6%). In contrast, reduced CYP2C9 metabolism was virtually absent in Ghana and Nigeria (Table 3).

**Table 3:** Frequencies of inferred CYP2C9 metabolizer phenotype

Country	NM (in %)	IM (in %)	PM (in %)
**Europe**			
*South Europe*			
Bulgaria	64.0	33.6	2.4
Croatia	54.8	41.2	4.0
Greek	62.4	34.9	2.8
Italy	61.2	35.6	3.2
North Macedonia	64.9	32.9	2.3
Portugal	62.1	35.2	2.8
Serbia	63.7	33.7	2.6
Spain	58.0	38.2	3.8
Turkey	63.5	33.5	3.0
*North Europe*			
Denmark	68.2	30.2	1.6
Estonia	71.1	27.1	1.7
Finland	67.8	30.4	1.8
Norway	69.9	28.4	1.7
Sweden	67.0	31.1	1.9
*Central and East Europe*			
Czech	68.2	30.1	1.7
Germany	65.6	32.7	1.7
Hungary	61.9	35.1	3.0
Romania	63.0	34.0	3.0
Russian	68.6	29.6	1.9
Slovak	67.2	30.5	2.2
Slovenia	66.4	31.6	1.9
*West Europe*			
Belgium	67.6	30.3	2.1
France	59.3	37.7	3.0
Netherlands	65.6	32.5	1.9
UK	70.8	27.7	1.5
**Africa**			
Benin	91.2	8.8	0.0
Egypt	67.2	31.0	1.8
Ethiopia	78.3	21.0	0.6
Gambia	99.2	0.8	0.0
Ghana	100.0	0.0	0.0
Morocco	81.9	18.1	0.0
Mozambique	64.2	35.5	0.4
Nigeria	100.0	0.0	0.0
Sudan	82.8	16.9	0.3
**Middle East**			
Iran	56.9	40.3	2.8
Israel	60.0	37.0	3.0
Jordan	63.5	34.2	2.3
Kuwaiti	65.6	32.7	1.7
Lebanon	59.0	38.0	3.0
Oman	79.9	19.5	0.6
Saudi Arabia	66.1	31.9	2.0
United Arab Emirates	40.2	48.7	11.1
**ASIA**			
*Southeast Asia*			
Indonesia	95.3	4.7	0.1
Malay	92.4	7.5	0.1
Thai	89.6	10.1	0.3
Vietnam	94.2	5.7	0.1
*East Asia*			
Bhutan	99.3	0.7	0.0
China	92.9	7.8	0.2
Japan	95.2	4.8	0.1
Korea	97.8	3.0	0.0
*South Asia*			
Bangladesh	75.2	23.1	1.7
Indian	74.3	24.0	1.7
Pakistani	69.0	28.4	2.6
**South America**			
Argentina	89.1	10.6	0.3
Bolivia	85.0	14.6	0.4
Brazil	62.6	35.2	2.1
Chile	75.7	23.4	0.9
Columbia	70.4	27.9	1.7
Ecuador	91.8	8.0	0.2
Peru	84.3	15.2	0.5
Uruguay	63.0	34.4	2.6
Venezuela	81.6	18.0	0.4
**North America**			
Costa Rica	85.5	14.1	0.3
Cuba	62.4	34.6	3.0
Mexico	87.6	12.2	0.3
**Oceania**			
Australia	64.5	33.3	2.2

NM, normal metabolizers; IM, intermediate metabolizers; PM, poor metabolizers

## Discussion

Genetic variations in *CYP2C9* are major determinants of impaired CYP2C9 enzyme activity. In this study, we presented country-specific frequencies of functionally important *CYP2C9* alleles at a global scale by integrating available genotype data from 108 studies comprising > 81,000 individuals in total. Our analysis showed that CYP2C9*2 is most prevalent in Middle Eastern populations (up to 18.1%), followed by South European populations (up to 16.5%), whereas CYP2C9*3 is most abundant in Emiratis (21.3%) and South Asian populations (up to 11.9%), followed by South European populations (up to 10.1%). These results are overall in accordance with previous studies that analyzed frequency data aggregated by continent or major ethnic groups [14, 15].

Our data indicate extensive variation within superpopulations, as evidenced by frequencies of CYP2C9*3 in the Jahai people that were up to tenfold higher than in geographically adjacent populations in Malaysia, Thailand and Vietnam. This population is considered to be among the earliest settlers entering the Malaysian peninsula from Africa over 50,000 years ago and endogamy results in a distinct genetic profile and high frequency founder mutations [20]. Similar results were observed for the Uygur population from Xinjiang, China, that harbors high frequencies of CYP2C9*2 (7.8%), an allele which is otherwise very rare in East Asia (< 0.1%). Previous studies showed that the Uygurs are a highly admixed population with admixture mapping suggesting a European ancestry contribution of 47% [21]. In South America, we observed large frequency differences of CYP2C9*2 (between 0.5% in Ecuador to 6% in Chile) but not *3 across different mestizo populations (Table 2). This is not surprising given the well-documented fluidity between Amerindian and European ancestral contributions [22] and similar results in different mestizo populations in Mexico [23].

Besides differences between ethnic groups, we also observed heterogeneity between allele frequencies of countries within the same macrogeographical region. For example, the frequency of CYP2C9*2 in Turkey (10.5%) were considerably lower than in its neighboring countries Bulgaria (12.5%), Greece (12.9%), Lebanon (15.4%) and Iran (18.1%). Similarly, CYP2C9*3 prevalence was graded across Europe with overall lower frequencies in North and Central Europe (4.7%-7.2%) compared to South and East Europe (7.9%-10.1%) in agreement with previous reports [24]. These results were corroborated by a large aggregate study in Scandinavia, which confirmed *3 frequencies of 6% in a large sample of 3,503 individuals from Norway, Sweden, Denmark and Finland [25]. Most pronounced differences were observed between the indigenous Tiwi population and groups of European ancestry in Australia with frequencies of CYP2C9*2 (12.8% and 0% in Europeans and Tiwi, respectively) and CYP2C9*3 (6.9% and 36%, respectively) differing by more than fivefold [26]. Genetic variability profiles of Tiwi were moreover drastically different from indigenous Polynesian and Maori populations. Substantial variability in prevalence of CYP2C9*2 (0.5–13%) and CYP2C9*3 (2.3–7.6%) was also observed across South America likely due to differing admixture between Amerindians, Europeans and Sub-Saharan Africans [27]. Therefore, genetic information of small geographically defined groups cannot provide accurate estimations of national allele frequencies particularly in countries with high population diversity [28]. Furthermore, our analyses conclude that there remains a pronounced underrepresentation of ethnogeographic groups, particularly in Central and Southeast Asia, Oceania and parts of Africa. Combined, these results demonstrate that pharmacogenomic studies require high ethnogeographic resolution to maximize its socioeconomic benefits, particularly for indigenous or founder populations with distinct genetic profiles.

Genetic profiles constitute important factors to infer metabolic phenotypes and *CYP2C9* genotypes were sufficient to correctly identify the majority of individuals with impaired CYP2C9 activity [29, 30]. Estimation of metabolizer status at the population-scale, requires the aggregation of prevalence data from as many as possible functionally relevant alleles. While distribution patterns of CYP2C9*2 and *3 have been extensively profiled, considerably less information is available about frequencies of alleles considered to be population-specific, such as, *5, *6, *8, *11 for Africans and *13, *14 for Asians. Consequently, the calculated IM and PM frequencies could be underestimated for countries with missing data. Besides star alleles, also rare variants without functional information can contribute to altered CYP2C9 metabolism and have been associated with hypersensitivity to CYP2C9 substrates [31]. Overall, rare variants that have not been assigned star alleles have been estimated to account for 3.4% of the genetically encoded functional variability in *CYP2C9* and could thus be an additional, but minor source of underestimation [18]. In addition, co-medication with inhibitors or inducers of CYP2C9 or impaired liver function can also affect CYP2C9 metabolic phenotypes, indicating that prescribers must integrate both genetic and non-genetic factors to guide treatment decisions.

CYP2C9 functionality is clinically relevant for a variety of commonly prescribed drugs. CYP2C9*2 and *3 have been associated with mean weekly warfarin dose [32] and carrier status of reduced function *CYP2C9* alleles impacts the risk of warfarin-induced bleeding complications [33–35]. Consequently, guidelines recommend the use of pharmacogenetically guided dosing algorithms that consider CYP2C9*2 and *3, with adjustments for patients with African ancestry based on carrier status of CYP2C9*5, *6, *8 and *11 [36]. Reduced CYP2C9 metabolizer status is furthermore linked to phenytoin related neurotoxicity [37] and our data indicate that up to 40% of patients in Southern Europe and the Middle East might benefit from maintenance dose reductions of 25–50% [38]. Furthermore, reduced CYP2C9 activity is associated with higher drug exposure and increased risk of gastrointestinal bleeding, hypertension and myocardial infarction upon treatment with multiple NSAIDs, including oxicams, celecoxib, flurbiprofen and ibuprofen [39–42]. The current treatment recommendations are to reduce starting doses in poor CYP2C9 metabolizers by 50–75% and to titrate upwards with caution after steady-state concentrations are reached NSAIDs [43]. Based on global *CYP2C9* allele distributions, these recommendations apply to more than 3% of patients in Croatia, Italy, Spain, France and Israel.

In order to implement *CYP2C9* genotyping in a clinical setting, it is essential to consider the cost-effectiveness of preemptive *CYP2C9* genotyping followed by treatment adjustment for decreased function allele carriers. For warfarin, previous cost-effectiveness analyses indicated that dosing guided by both *CYP2C9* and *VKORC1* genotypes was likely to be cost-effective compared to conventional dosing regimens [44, 45]. We are not aware of studies that evaluate the cost-effectiveness of preemptive *CYP2C9* genotyping to guide therapy with phenytoin or NSAIDs. However, previous studies have shown that allele frequencies are one of the most important determinants of the cost-effectiveness of preemptive pharmacogenomic testing at the national level [46]. The data presented here might thus be useful for policy makers to evaluate whether *CYP2C9* genotyping, most likely in a panel together with variants in other genes, might provide added value for national healthcare systems.

## Conclusions

In summary, this study presents the worldwide distribution of *CYP2C9* alleles and inferred metabolizer phenotypes with high ethnogeographic resolution. The results reveal global patterns as well as unexpected disparities of *CYP2C9* genotype variability. Reduced CYP2C9 activity is most prevalent in South Europe and the Middle East, as well as in specific founder populations in Southeast Asia. The data presented here can serve as a valuable resource for population-specific CYP2C9 allele and phenotype frequencies that can provide important information for the guidance of personalized drug therapy and inform precision public healthcare at the global scale.

## Methods

### Data sources

We performed a systematic literature search in PubMed database covering publications before February 2023. All studies reporting frequencies of CYP2C9*2 (rs1799853) and CYP2C9*3 (rs1057910) in defined populations with cohort sizes ≥ 50 were included. We also included studies reporting frequencies of the functional alleles *5 (rs28371686), *6 (rs9332131), *8 (rs7900194), *11 (rs28371685), *13 (rs72558187) and *14 (rs72558189). In addition to published studies, we included population frequency data from the Genome Aggregation Database [47] and the 1000 Genomes Project [48]. As a result, we identified a total of 108 original articles reporting studied *CYP2C9* allele frequencies from 81,662 unrelated individuals using a variety of methods for variant detection (Additional file 1: Table S1 and Additional file 2: Table S2). Frequency data for countries and ethnogeographic groups were aggregated using a weighted average approach using the cohort sizes as weighting factors.

### Phenotype analyses

*CYP2C9* allele function was defined based on the PharmVar consensus classifications [16]. CYP2C9 metabolizer phenotypes were defined according to the CPIC guideline [43]. Phenotype assignment of diplotypes is provided in Additional file 3: Table S3. Based on these definitions, frequencies of analyzed *CYP2C9* alleles, i.e., decreased function allele *2, *5, *8, *11, *14 and LOF allele *3, *6 and *13, were used to calculate phenotype frequencies based on the Hardy–Weinberg equation. Frequencies of the *CYP2C9* reference allele (*1) were calculated as f_*1_ = 1 − Σ_i_ *f*_i_, with *f*_i_ being the frequency of each analyzed variant allele *i*.

## Supplementary Information

**Additional file 1**. National frequencies of other six functionally relevant *CYP2C9* variant alleles.

**Additional file 2**. Original articles reporting *CYP2C9* allele frequencies.

**Additional file 3**. CYP2C9 metabolizer phenotypes defined by different diplotypes.

## Abbreviations

ADME: Administration, distribution, metabolism and excretion
ADR: Adverse drug reaction
CPIC: The Clinical Pharmacogenetics Implementation Consortium
CYP: Cytochrome P450
EMA: The European Medicines Agency
FDA: The US Food and Drug Administration
IM: Intermediate metabolizer
LOF: Loss-of-function
MAF: Minor allele frequency
NM: Normal metabolizer
NSAID: Non-steroidal anti-inflammatory drug
PM: Poor metabolizer
